# Being a psychiatric resident during COVID times – personal experiences of Hungarian trainees

**DOI:** 10.1192/j.eurpsy.2022.594

**Published:** 2022-09-01

**Authors:** C. Asbóth, E. Gergics, S. Gurzó, A. Herczeg, A. Hrapcsák, F. Kupcsik, P. Nagy, O. Oláh, G. Szilvágyi, P. Szocsics, Z. Szűcs, I. Bitter

**Affiliations:** 1 Semmelweis University, Department Of Psychiatry And Psychotherapy, Budapest, Hungary; 2 Péterfy Hospital and Manninger Jenő National Institute of Traumatology, Department Of Psychiatry And Crisis Intervention, Budapest, Hungary; 3 Szent György University Teaching Hospital, Department Of Psychiatry, Székesfehérvár, Hungary; 4 Jahn Ferenc South-Pest Hospital, Department Of Psychiatry, Budapest, Hungary; 5 National Institute of Mental Health, Neurology and Neurosurgery, Nyírő Gyula Hospital, Budapest, Hungary; 6 Saint John Hospital, Family Centred Mental Health Centre, Budapest, Hungary; 7 Szent Borbála Hospital, Department Of Psychiatry And Addictology, Tatabánya, Hungary

**Keywords:** residency training, psychiatry, personal experiences, Covid-19

## Abstract

**Introduction:**

During the COVID-19 pandemic residents of the central region of Hungary also had to adapt to several challenges such as changes of hospitals’ specialty profiles and delegation of health care workers to COVID wards.

Hungarian residents have their practical training in various hospitals, while their psychiatric academic training is organised in groups.

**Objectives:**

Our aim is to share our personal experiences about how our work and training have changed during the pandemic and it’s effect on our patients.

**Methods:**

Participants of the study were the authors of the poster. Responses to open questions were structured based on the following topics: competencies in internal medicine, infectious diseases and psychiatry, our collaboration with other medical disciplines, psychiatric training and attitudes towards mental health patients.

**Results:**

We worked min 2 weeks max 8 months at COVID wards and also treated COVID-19 infected psychiatric patients, thus gaining a greater experience in general medicine. In psychiatric work, acute care became prominent, communication in PPE and restricted contact with patients’ relatives were particularly difficult. Our relationship with other specialists has improved, consultation became easier. Increased use and misuse of psychiatric consultation requests led to further pressure. Restrictions, stigmatisation and discrimination increased against psychiatric patients, including difficult access to care. Psychiatric training in the hospitals became limited, however seminars organized by the university continued online with our active participation.

**Conclusions:**

During the pandemic we gained greater experience in general medicine. Psychiatric care and our training was negatively affected, however the latter was mitigated by online seminars.

**Disclosure:**

No significant relationships.

